# Inhibition or Knockdown of ABC Transporters Enhances Susceptibility of Adult and Juvenile Schistosomes to Praziquantel

**DOI:** 10.1371/journal.pntd.0003265

**Published:** 2014-10-16

**Authors:** Ravi S. Kasinathan, Lalit Kumar Sharma, Charles Cunningham, Thomas R. Webb, Robert M. Greenberg

**Affiliations:** 1 Department of Pathobiology, School of Veterinary Medicine, University of Pennsylvania, Philadelphia, Pennsylvania, United States of America; 2 Department of Chemical Biology and Therapeutics, St. Jude Children's Research Hospital, Memphis, Tennessee, United States of America; 3 Department of Biology, University of New Mexico, Albuquerque, New Mexico, United States of America; 4 SRI International, Menlo Park, California, United States of America; Uniformed Services University, United States of America

## Abstract

Parasitic flatworms of the genus *Schistosoma* cause schistosomiasis, a neglected tropical disease that affects hundreds of millions. Treatment of schistosomiasis depends almost entirely on the drug praziquantel (PZQ). Though essential to treating and controlling schistosomiasis, a major limitation of PZQ is that it is not active against immature mammalian-stage schistosomes. Furthermore, there are reports of field isolates with heritable reductions in PZQ susceptibility, and researchers have selected for PZQ-resistant schistosomes in the laboratory. P-glycoprotein (Pgp; ABCB1) and other ATP binding cassette (ABC) transporters remove a wide variety of toxins and xenobiotics from cells, and have been implicated in multidrug resistance (MDR). Changes in ABC transporter structure or expression levels are also associated with reduced drug susceptibility in parasitic helminths, including schistosomes. Here, we show that the activity of PZQ against schistosome adults and juveniles *ex vivo* is potentiated by co-administration of either the highly potent Pgp inhibitor tariquidar or combinations of inhibitors targeting multiple ABC multidrug transporters. Adult worms exposed to sublethal PZQ concentrations remain active, but co-administration of ABC transporter inhibitors results in complete loss of motility and disruption of the tegument. Notably, juvenile schistosomes (3–4 weeks post infection), normally refractory to 2 µM PZQ, become paralyzed when transporter inhibitors are added in combination with the PZQ. Experiments using the fluorescent PZQ derivative (*R*)-PZQ-BODIPY are consistent with the transporter inhibitors increasing effective intraworm concentrations of PZQ. Adult worms in which expression of ABC transporters has been suppressed by RNA interference show increased responsiveness to PZQ and increased retention of (*R*)-PZQ-BODIPY consistent with an important role for these proteins in setting levels of PZQ susceptibility. These results indicate that parasite ABC multidrug transporters might serve as important targets for enhancing the action of PZQ. They also suggest a potentially novel and readily-available strategy for overcoming reduced PZQ susceptibility of schistosomes.

## Author Summary

Schistosomes are parasitic flatworms that cause schistosomiasis, a tropical disease affecting hundreds of millions worldwide. Praziquantel (PZQ) is the current drug of choice against schistosomiasis, and, indeed, is the only approved antischistosomal treatment available in most parts of the world. Though effective overall, PZQ has limitations, including its lack of activity against immature schistosomes. Furthermore, reported cure rates in the field are often below optimal levels, and there is increasing evidence that schistosomes can become resistant to the drug. ABC transporters such as P-glycoprotein are efflux transporters that mediate detoxification of cells via removal of toxins and xenobiotics, including drugs. They underlie multidrug resistance in mammalian cells, and are also associated with drug resistance in parasitic worms, including schistosomes. Here, we show that compounds that inhibit these efflux transporters potentiate the activity of PZQ against schistosomes, including normally PZQ-insensitive juvenile worms. Similarly, suppressing expression of these transporters also increases adult worm responsiveness to PZQ. Our experiments may provide insights into the role of these drug transporters in PZQ action, and could also translate into new therapeutic strategies for augmenting treatment of schistosome infections and overcoming drug resistance.

## Introduction

Schistosomiasis is a highly prevalent tropical disease caused by parasitic flatworms of the genus *Schistosoma*. It is widespread, affecting hundreds of millions throughout the tropics and sub-tropics, with devastating effects on human health and economic development. Estimates suggest that schistosomiasis is responsible for almost 300,000 deaths annually in sub-Saharan Africa alone [1], [2], [3].

In the absence of an effective vaccine, chemotherapeutic intervention remains the main means of treating and controlling schistosomiasis. The current drug of choice is praziquantel (PZQ). PZQ is effective against all human schistosome species and has relatively mild side effects [4], [5], [6]. It has proved successful in large-scale control efforts targeting schistosomiasis in several countries [7], [8] and, as a result of its advantages and reduced costs, has become the only available antischistosomal treatment in most parts of the world [5], [9]. Unfortunately, reliance on a single drug for a disease of this magnitude is dangerous [6], and is particularly so for PZQ since the mode of action is not rigorously defined [10], [11], [12]. Additionally, reported failure rates for PZQ in the field are typically in the range of 30–50% [13], [14], [15], and several field isolates [4], [16] and experimentally-induced, drug-selected schistosomes [17], [18] exhibit reduced susceptibility to the drug, perhaps a harbinger for the emergence and ultimate spread of PZQ resistance [reviewed in 12], [19], [20]. Schistosomes also show major stage-specific differences in PZQ susceptibility; immature worms are refractory to PZQ, making treatment largely ineffective until approximately 5–6 weeks post-infection [21], [22], [23], [24].

One strategy proposed to enhance drug efficacy and overcome drug resistance is to augment current anthelmintics with agents targeting different, but potentially interacting sites of action, including cellular components that regulate rates of drug uptake, metabolism, or efflux. Efflux transporters that mediate multidrug resistance have been advanced as particularly appealing targets of this type [25], [26].

P-glycoprotein (Pgp; ABCB1) and related efflux transporters such as multidrug resistance associated protein 1 (MRP1; ABCC1) and breast cancer resistance protein (BCRP; ABCG2) are members of the ATP binding cassette (ABC) superfamily of proteins. ABC transporters use energy generated from ATP hydrolysis to translocate compounds across the membrane. They typically exhibit broad substrate specificity, with their most obvious physiological role being to remove or exclude metabolic toxins and xenobiotics, including therapeutic agents, from cells and tissues. However, in addition to this detoxification activity, ABC transporters can transport a variety of biologically significant signaling molecules with high affinity [27], [28], [29], [30], and have been implicated in a wide array of physiological functions [31], [32], [33], including modulation of cell death pathways [34], [35] and immune function [36].

Most notably, ABC multidrug transporters underlie multidrug resistance, a phenomenon in which cells that develop resistance to a particular drug show simultaneous cross-resistance to several structurally unrelated compounds [37]. ABC transporters have additionally been implicated in insusceptibility to anthelmintics, as changes in expression levels and allele frequencies of Pgp and other ABC transporters are found in parasitic helminths exhibiting reduced drug sensitivity [reviewed in 38], [39], [40], [41].

Like mammals, schistosomes have genes for a wide variety of ABC transporters, including Pgp, MRP1, and BCRP [41], [42]. Previous work has focused on particular *S. mansoni* orthologs of Pgp (SMDR2) and MRP1 (SmMRP1), and the role they may play in the parasite's physiology and susceptibility to PZQ. For example, *S. mansoni* upregulate expression of SMDR2, SmMRP1, and other drug transporter RNAs and anti-Pgp and anti-MRP1 immunoreactivity in response to sub-lethal concentrations of PZQ [43], [44], [45]. Furthermore, some adult worms with reduced susceptibility to PZQ exhibit higher basal levels of these transporters [43], [44], and PZQ interacts directly with expressed recombinant SMDR2, as both an inhibitor and a likely substrate [46]. Our work has also implicated these transporters in schistosome reproduction [47], while others have demonstrated likely involvement of these transporters in parasite excretory activity [48], [49].

Here, we show that disruption of schistosome ABC transporter function (by pharmacological inhibition) or expression (by RNA interference) can potentiate the antischistosomal activity of PZQ against adult worms in culture, appearing to increase the effective intraworm concentration of PZQ. Remarkably, co-administration of MDR inhibitors with PZQ also renders PZQ-insusceptible juvenile schistosomes susceptible to PZQ. Based on these findings, as well as those discussed above, we hypothesize that schistosome ABC transporters modulate the responsiveness of schistosomes to PZQ. These results also suggest that augmentation of standard PZQ therapy with readily-available inhibitors of Pgp or other multidrug transporters has the potential to enhance drug efficacy and possibly prevent emergence or spread of PZQ resistance.

## Results

### Inhibitors of Pgp and other ABC multidrug transporters increase susceptibility of adult *S. mansoni* to PZQ

In these experiments, we tested whether inhibitors of ABC multidrug transporters could potentiate the activity of sub-lethal concentrations of PZQ against adult schistosomes *ex vivo*. Worms were incubated in PZQ alone (500 nM) or in PZQ combined with the ABC transporter inhibitors for 12–24 hours, removed to drug-free medium, and then tested for recovery of motility following 48 hours in drug-free medium.

**Figure 1:**
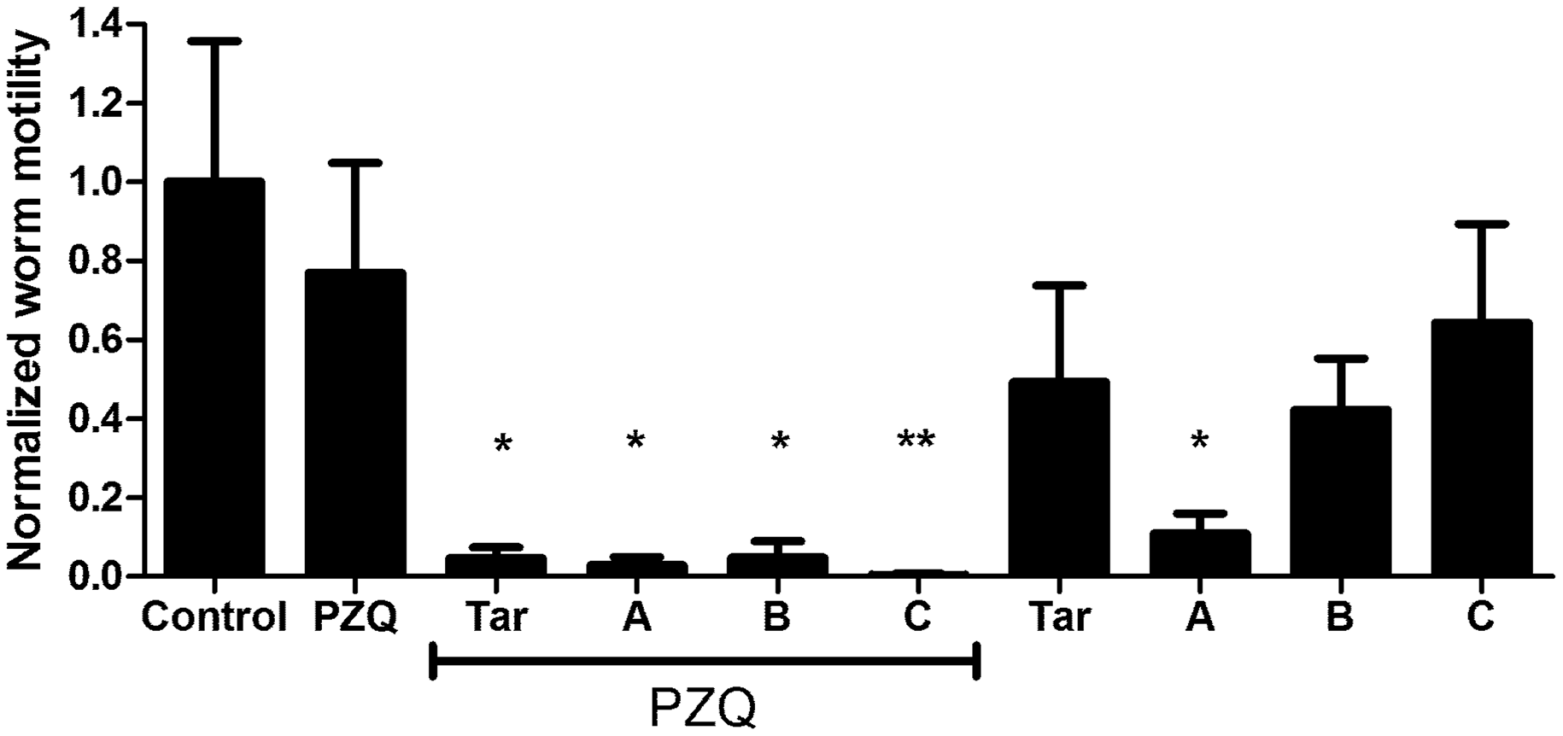
ABC transporter inhibitors enhance susceptibility of adult *S. mansoni* to PZQ. Adult parasites were perfused at 6–7 weeks post-infection and incubated overnight in schistosome medium containing the compounds as noted. Following 48 h recovery in media alone, worm motility was assessed in individual worms using a video camera and quantifying change in distal/proximal distance using MaxTraqLite+ software. Values were normalized to control worms, as described in Materials and Methods. Control worms were incubated in 0.5% DMSO (n = 7). PZQ = 500 nM PZQ (n = 9); Tar = 10 µM tariquidar (n = 7 alone; n = 7 plus PZQ); A = Combination A (10 µM zosuquidar, 10 µM Ko143, 25 µM MK 571; n = 5 alone; n = 4 plus PZQ); B = Combination B (10 µM elacridar, 20 µM Reversan; n = 8 alone; n = 6 plus PZQ); C = Combination C (20 µM dexverapamil, 25 µM MK 571; n = 7 alone; n = 8 plus PZQ). Labels underscored by the PZQ line included 500 nM PZQ as well. *, ** indicate P<0.05 and P<0.01, respectively, compared to control worms, ANOVA with Dunnett's Multiple Comparison post test.

*S. mansoni* adults exposed to various ABC multidrug transporter inhibitors in combination with 500 nM PZQ exhibit significant loss of motility compared to those exposed to PZQ alone. Tariquidar (XR9576), a third-generation, highly potent Pgp inhibitor [50], [51], [52], [53], is particularly effective (Fig. 1); inclusion of 10 µM tariquidar with 500 nM PZQ results in essentially complete loss of detectable schistosome motility. In contrast, worms in PZQ alone remained highly active. Other inhibitors were effective at potentiating PZQ activity in combinations that block different classes of ABC transporters (combinations A, B, C; see Materials and Methods). Thus, Combination A includes three compounds and Combination B includes two compounds that inhibit three classes of mammalian transporters (Pgp, MRP1, and BCRP); Combination C contains inhibitors of two classes of mammalian transporters (Pgp and MRP1). All of these inhibitor combinations have significant effects on adult schistosome motility when combined with 500 nM PZQ. Interestingly, Combination A (zosuquidar, Ko143, MK 571) also significantly suppresses worm motility on its own (Fig. 1).

**Figure 2:**
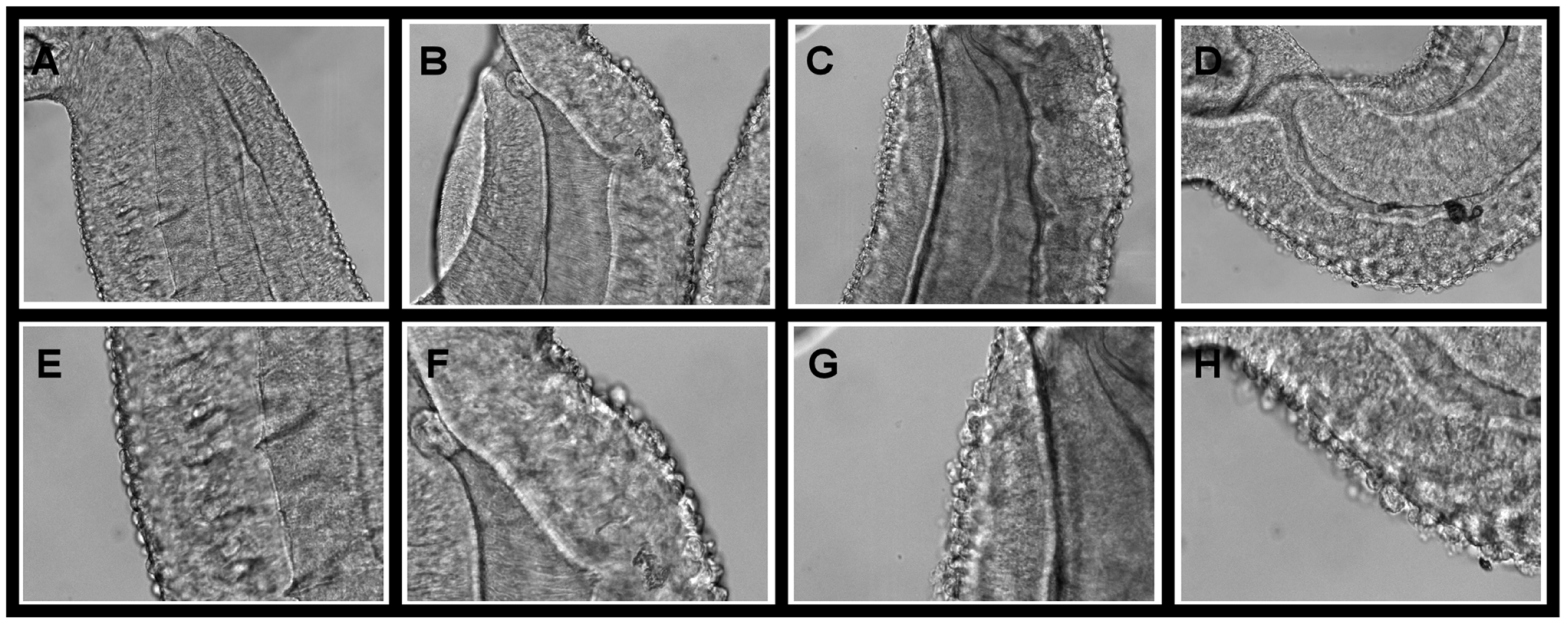
ABC transporter inhibitors increase PZQ-induced tegumental disruption in adult worms. Adult schistosomes were treated as in Fig. 1, and male worms analyzed by optical microscopy in an equivalent tegumental region just below the oral sucker. **A–D** show the overall region analyzed; **E–H** are zoomed images of equivalent regions of **A–D**, with damage more apparent. (**A, E**) 500 nM PZQ. (**B, F**) 500 nM PZQ+Combination A. (**C, G**) 500 nM PZQ+Combination B. (**D, H**) 500 nM PZQ+10 µM tariquidar.

In addition to dramatic effects on adult worm motility, PZQ also causes disruption of the tegument [54]. ABC multidrug inhibitors also potentiate these effects of PZQ on tegumental integrity. Thus, as shown in Fig. 2, adult schistosomes incubated in the relatively low PZQ concentration of 500 nM show little if any indication of the type of tegumental disruption seen with higher levels of PZQ. In contrast, those schistosomes co-incubated in PZQ plus the ABC transporter inhibitors display significant tegumental damage, with the normal tubercular surface showing multiple blebs and lesions. Interestingly, 10 µM tariquidar appears also to produce some damage to the tegument in the absence of PZQ (Fig. S1), perhaps signifying potential effectiveness as an antischistosomal on its own. Though we have not quantified the level of damage, visual inspection suggests that PZQ plus tariquidar produces a more severe effect than tariquidar alone. In contrast, no obvious effects on the tegument are apparent when worms are incubated without PZQ in Combinations A, B, or C.

### Inhibitors of Pgp and other ABC multidrug transporters increase retention of BODIPY-PZQ fluorescence in adult *S. mansoni*

We have used (*R*)-PZQ-BODIPY [21], [46], a fluorescent derivative of the active enantiomer [55], [56], [57], [58] of PZQ, to explore the mechanism by which ABC transporter inhibitors might be enhancing the effective potency of PZQ on adult schistosomes. To investigate whether transporter inhibition increases retention of BODIPY-PZQ-linked fluorescence, we exposed adult schistosomes to 1 µM (*R*)-PZQ-BODIPY in the presence or absence of ABC transporter inhibitors. Following an overnight incubation, the medium was replaced with medium containing the same ABC transporter inhibitors (if any), but not the fluorescent PZQ. As controls, we also examined the effects of the transporter inhibitors on their own; some of these inhibitors (eg, tariquidar) increased worm fluorescence in the absence of (*R*)-PZQ-BODIPY, and were therefore excluded from further analysis.

**Figure 3:**
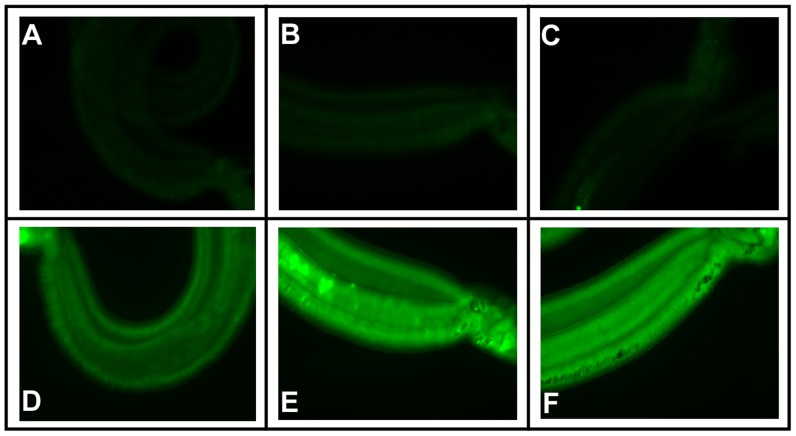
The ABC transporter inhibitors dexverapamil and MK 571 increase fluorescence in adult male worms exposed to (*R*)-PZQ-BODIPY. Fluorescent microscopy of adult male schistosomes. Worms were incubated in the designated compounds as described in Materials and Methods. (**A**) DMSO control (no drugs). (**B**) 10 µM dexverapamil. (**C**) 10 µM MK 571. (**D**) 1 µM (*R*)-PZQ-BODIPY. (**E**) 1 µM (*R*)-PZQ-BODIPY+10 µM dexverapamil. (**F**) 1 µM (*R*)-PZQ-BODIPY+10 µM MK 571. All images were taken with identical exposures.

**Figure 4:**
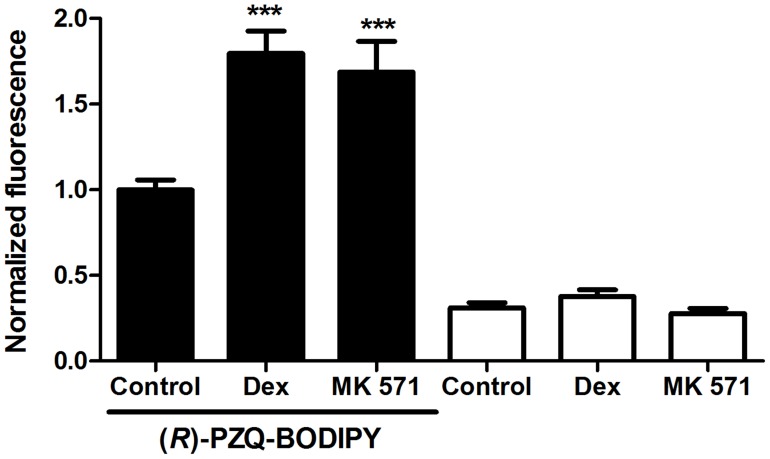
Dexverapamil and MK 571 significantly increase measured fluorescence intensity in adult male worms exposed to (*R*)-PZQ-BODIPY. Schistosomes were exposed to 1 µM (*R*)-PZQ-BODIPY in the presence or absence of 10 µM dexverapamil or 10 µM MK 571, as described, and Image J was used to measure fluorescence intensity from images taken at identical magnification and exposure levels. Intensity measurements on all worms were from an identically-sized rectangular area in an equivalent region of the worm slightly below the oral sucker. Intensity values were normalized to the (*R*)-PZQ-BODIPY samples. Data are from measurements of several worms spanning two independent experiments. Black bars represent worms incubated in (*R*)-PZQ-BODIPY, with added dexverapamil (Dex; n = 10), MK 571 (n = 10), or DMSO carrier (Control; n = 11); white bars represent worms incubated without (*R*)-PZQ-BODIPY, but with dexverapamil (Dex; n = 7), MK 571 (n = 7), or DMSO carrier (Control; n = 11). *** indicates P<0.001 compared to (*R*)-PZQ-BODIPY Control, ANOVA with Dunnett's Multiple Comparison post test.

Adult schistosomes exhibit some green autofluorescence, but incubation in (*R*)-PZQ-BODIPY produces a dramatic increase in green fluorescence within worms (Fig. 3). The intensity of this diffuse fluorescence within males appears to increase dramatically when either the Pgp inhibitor dexverapamil or the MRP1 inhibitor MK 571 is included with the (*R*)-PZQ-BODIPY (Fig. 3). Male worms exposed to both (*R*)-PZQ-BODIPY and either of these ABC transporter inhibitors exhibit a significant 1.7–1.8-fold increase in fluorescence intensity compared to worms exposed to (*R*)-PZQ-BODIPY alone (Fig. 4). It is possible that inhibition of the schistosome transporters blocks efflux of the BODIPY moiety of BODIPY-PZQ rather than (or in addition to) the PZQ moiety. However, worms incubated in CellTracker BODIPY, a membrane-permeable form of BODIPY that is modified intracellularly to a membrane-impermeable form, showed no significant difference in fluorescence intensity between worms incubated with or without dexverapamil or MK 571 (Fig. S2).

### Inhibitors of Pgp and other ABC multidrug transporters increase susceptibility of juvenile *S. mansoni* to PZQ

**Figure 5:**
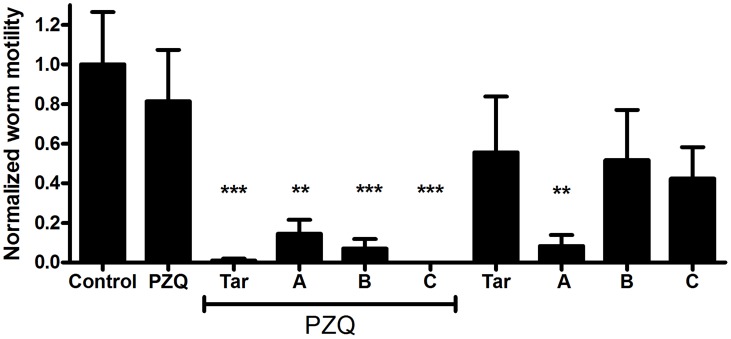
ABC transporter inhibitors enhance susceptibility of juvenile *S. mansoni* to PZQ. Immature worms (3–4 weeks post infection) were incubated in media plus the compounds as noted, and motility analyzed as in Fig. 1. Worms were incubated overnight in schistosome medium containing the compounds as noted, and allowed to recover for 72 h in schistosome medium alone. Compounds and combinations are as described in Fig. 1. Control (n = 6); PZQ (n = 8); Tar (n =  4 alone; n = 7 plus PZQ); Combination A (n = 5 alone; n = 7 plus PZQ); Combination B (n = 6 alone; n = 10 plus PZQ); Combination C (n = 6 alone; n = 7 plus PZQ). ** and *** indicate P<0.01 and P<0.001 respectively, compared to Control, ANOVA with Dunnett's Multiple Comparison post test.

Juvenile (3–4 week post infection) *S. mansoni* are refractory to PZQ, both *ex vivo* and *in vivo* [21], [22], [23], [24]. In these experiments, we examined whether inhibitors of Pgp and other ABC transporters could potentiate the activity of PZQ against juvenile *S. mansoni*, rendering those worms susceptible to the drug. Schistosomes were incubated overnight in 2 µM PZQ. As reported by others [24], while in PZQ they exhibit limited motility and show signs of contractile paralysis. However, following subsequent incubation for up to 72 h in the absence of PZQ, these worms recover completely, and exhibit high levels of motility (Fig. 5). In contrast, schistosomes incubated in both 2 µM PZQ and ABC transporter inhibitors do not recover, and instead continue to remain paralyzed after recovery in the absence of PZQ and inhibitors (Fig. 5). Thus, juvenile worms exposed during the initial incubation period to 10 µM tariquidar or transporter inhibitor combinations A, B, or C in addition to PZQ show significant reductions in worm motility. Drug Combination A (zosuquidar, Ko143, MK 571), which disrupted adult motility on its own, also appeared to paralyze juvenile schistosomes, while tariquidar and Combinations B and C had no significant effects on juvenile schistosome motility in the absence of PZQ.

### Suppression of parasite ABC transporter expression increases the responsiveness of adult *S. mansoni* to PZQ and the retention of BODIPY-PZQ fluorescence

**Figure 6:**
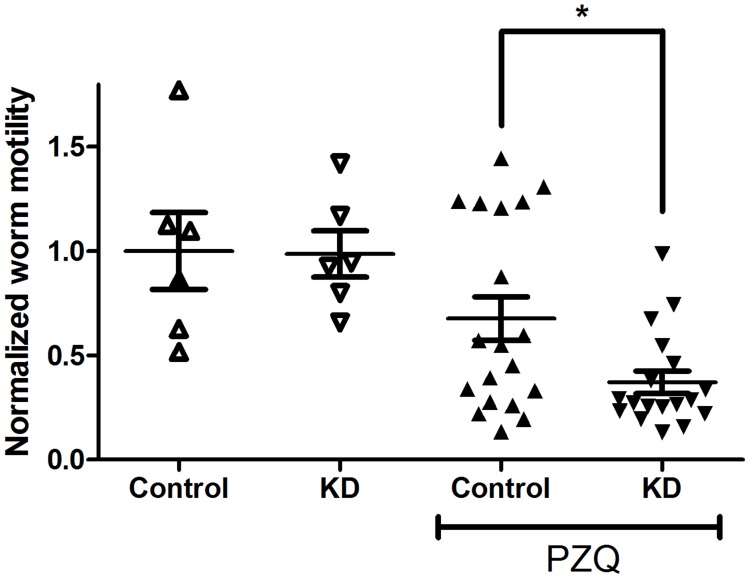
Suppression of *S. mansoni* ABC transporter expression enhances responsiveness of adult worms to PZQ. Adult schistosomes were electroporated with siRNAs targeting luciferase (Control) or the *S. mansoni* ABC transporters SMDR2, SmMRP1, ABCA4, ABCB6, and MRP7/ABCC10 (KD), as described in Materials and Methods. Following electroporation, worms were incubated in schistosome medium for 2 days, and then sorted into 2–3 worm pairs per well in a 12-well plate. They were then incubated in medium alone plus 0.5% DMSO, or in medium plus 800 nM PZQ, as described in Fig. 1, and subsequently analyzed for motility. n = 6 for the experiments without PZQ and n = 18 (KD) or 19 (Control) for the experiments in PZQ. Motility was measured as in Fig. 1, and results for individual worms are plotted. ** indicates P<0.01, ANOVA with Dunnett's Multiple Comparison post test.

We used siRNAs designed against different *S. mansoni* ABC transporters to knock down expression of these genes. Initial experiments in which we suppressed expression of individual transporters failed to enhance susceptibility of adult schistosomes to PZQ consistently. We subsequently tested simultaneous knockdown of multiple transporters. In these experiments, expression of the different target RNAs (SMDR2, SmMRP1, ABCA4, ABCB6, and ABCC10/MRP7; see Materials and Methods for further details) was reduced by 25–75% (Fig. S3). Adults in which expression of these genes is suppressed by RNAi are significantly less motile in 800 nM PZQ than control worms (Fig. 6). In contrast, there are no significant effects of knockdown on worm motility in the absence of PZQ, indicating that the suppression of transporter expression is not in itself reducing motility of these worms, but is rather decreasing their ability to counter the effects of PZQ. We have observed similar results upon knockdown of the four transporters, SMDR2, SmMRP1, ABCA4, and ABCB6.

**Figure 7:**
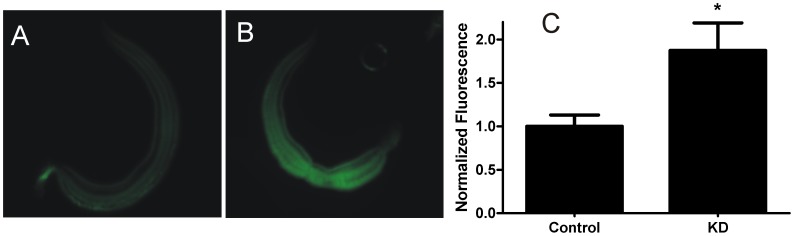
Suppression of *S. mansoni* ABC transporter expression in adult males increases retention of (*R*)-PZQ-BODIPY. Adult schistosomes were electroporated with siRNAs targeting luciferase (Control) or the *S. mansoni* ABC transporters SMDR2, SmMRP1, ABCA4, and ABCB6 (KD), as described in Fig. 6. Males were then exposed to 1 µM (*R*)-PZQ-BODIPY overnight. (**A**) Control worm electroporated with luciferase siRNA. (**B**) Worm electroporated with siRNAs targeting the ABC transporters. Both images were acquired with identical exposures and magnifications. (**C**) Normalized integrated fluorescence intensity for whole worms. Integrated intensity of fluorescence was quantified by designating the entire worm as the region of interest and subtracting mean background intensity, as described [80]. n = 5 worms for each condition. * indicates P<0.05, two-tailed t-test.

Similar to the effects of ABC transporter inhibitors described above, knockdown of expression of these transporters also increases retention of (*R*)-PZQ-BODIPY within the worm (Fig. 7). Thus, worms in which four ABC transporters (SMDR2, SmMRP1, ABCA4, ABCB6) have been targeted by siRNAs show significantly higher intraworm BODIPY-PZQ fluorescence than control worms exposed to luciferase siRNA. These results indicate that reduced transporter expression, like transporter inhibition, can increase effective concentrations of PZQ (or at least BODIPY-PZQ) within the parasite.

## Discussion

In the absence of effective vaccines, chemotherapy continues to be the major strategy for treatment and control of infections by schistosomes. Currently, PZQ is effectively the only drug available for treatment of a disease affecting hundreds of millions, a situation with the potential for disastrous consequences. Furthermore, although PZQ is effective overall, it does have limitations. Only certain schistosome stages are sensitive to PZQ, and reductions in parasite prevalence and transmission following drug treatment are often less than optimal. Typical 30% treatment failure rates are seen in the field, with up to 50% failure reported [13], [14], [15]. Even these numbers may be optimistic, as the standardly-used Kato-Katz technique for measuring schistosome egg counts is not reliable and tends to underestimate infection levels [59], [60], [61]. The threat of emerging drug resistance also represents an ever-present concern, particularly in mass drug administration programs.

Here, we show that inhibition or reduced expression of *S. mansoni* ABC multidrug transporters increases the susceptibility of both adult and immature schistosomes to PZQ. ABC multidrug transporters underlie multidrug resistance in mammalian cells, and have been linked to drug resistance in parasites, including helminths. We have hypothesized [41] that the efflux activity of Pgp and other parasite ABC multidrug transporters may serve to protect the parasite against PZQ. Previously, we showed that higher expression levels of schistosome ABC transporters are associated with reduced susceptibility of worms to PZQ [43], [44]. Furthermore, PZQ inhibits and is likely transported by *S. mansoni* Pgp (SMDR2) [46]. These two observations suggest a mechanism by which reduced ABC transporter expression or disrupted function might enhance schistosome susceptibility to PZQ. Thus, part of the parasite's defense against PZQ may include efflux of the drug via these transporters. Indeed, if some variant of the “hydrophobic vacuum” model for Pgp [62], [63] is correct, parasite Pgp and similar transporters may in fact be preventing PZQ from crossing the cell membrane, translocating it to the extracellular space from the lipid bilayer. Reducing transporter activity or levels may thereby increase effective dose and effectiveness. On the other hand, increased expression (or activity) of schistosome ABC transporters may serve to reduce susceptibility, and could perhaps represent a mechanism underlying development or maintenance of PZQ resistance. Our results showing that transporter inhibitors increase both PZQ susceptibility and BODIPY-PZQ fluorescence in worms are consistent with this hypothesis.

On its own, the third-generation, highly potent Pgp inhibitor tariquidar [50], [51], [52], [53] consistently and significantly increased susceptibility to PZQ in both juvenile and adult worms. In contrast, all the other inhibitors we tested increased PZQ susceptibility only when combined with other compounds that act on ABC transporters of other classes, possibly indicating functional redundancy in the ability of these transporters to protect the parasite against PZQ. Interestingly, tariquidar also appears to be a substrate of BCRP at low concentrations and a BCRP inhibitor at higher (>100 nM) concentrations [64]. On the other hand, elacridar, a compound which inhibits both Pgp and BCRP in mammals with sub-micromolar affinity [65], [66], did not increase PZQ susceptibility in a consistent manner unless used in combination with a mammalian MRP1 inhibitor (Reversan). This apparent requirement that multiple schistosome ABC transporter types must be blocked to enhance PZQ potency (with the exception of tariquidar) was surprising, as a single Pgp reversing agent (eg, racemic-, or R(+)-verapamil) has been reported to enhance activity of anthelmintics against other trematodes [67], as well as nematodes [40]. Furthermore, a single Pgp or MRP1 inhibitor can disrupt egg production in schistosomes and other flatworms [47], [68]. However, it is clear from the *S. mansoni* and *S. japonicum* genomes that there is a high level of redundancy in ABC transporter genes [41], and it is possible that the pharmacological sensitivities of some schistosome ABC transporters differ from those of their mammalian counterparts. Notably, PZQ appears to be a substrate for one *S. mansoni* transporter, the Pgp homolog SMDR2 [46]; in contrast, PZQ is not a substrate for mammalian Pgp, though it is an inhibitor [69]. Interestingly, single inhibitors (*eg*, dexverapamil, MK 571) increase intra-worm fluorescence following exposure of adult schistosomes to (*R*)-PZQ-BODIPY, indicating that blocking a single class of transporters can raise effective PZQ concentrations within the worm. The precise mechanism by which these intraworm PZQ concentrations might be enhanced, and the specific sites of PZQ retention within the parasite, are worthy of further investigation.

It is of course possible that the effects of the ABC transporter inhibitors on PZQ susceptibility are non-specific. In order to test that possibility, we performed experiments to determine whether knocking down levels of transporter gene expression also increases parasite susceptibility to PZQ. We found that simultaneous knockdown of schistosome ABC transporters from different classes could indeed render worms more susceptible to PZQ. The requirement for knockdown of multiple transporters to enhance PZQ susceptibility may reflect the redundancy in these genes discussed previously, as well as the limited efficiency (25–75%) of knockdown. Reduced expression of ABC transporters appears to have no measurable effect on motility of adult worms unless those worms are also exposed to PZQ, suggesting that the knockdown is not simply compromising the health of the parasite, but is instead having a more specific effect on how the parasite responds to PZQ, an interpretation supported by the increased retention of (*R*)-PZQ-BODIPY in these worms. It is also possible that disrupting expression or function of these transporters could interfere with the worm's ability to respond to other drugs and stressors, and might therefore represent a general strategy for potentiating activity of these agents against the parasite.

One interpretation of our results might be that combination of a single ABC transporter inhibitor (other than tariquidar) with PZQ might not augment PZQ antischistosomal action in real-world situations, and tariquidar, an experimental and expensive compound, is clearly not a realistic option. However, it will be important to determine whether the results we have obtained *in vitro* are predictive for schistosomes *in vivo*, where host factors that could increase parasite vulnerability might come into play. Furthermore, we and others have shown that single ABC transporter inhibitors disrupt schistosome egg production *in vitro* and similarly decrease parasite egg burden and resultant pathology *in vivo* [47], [68]. Should PZQ resistance arise, these inhibitors - many of which are approved, safe, and inexpensive - might be combined with PZQ in a strategy to reduce spread of those resistant worms by reducing egg production and transmission of resistant parasites. Such a strategy could be exploited as a multifaceted approach to reduce morbidity, disease transmission, and spread of resistance [70].

## Materials and Methods

### Ethics statement

This study was carried out in strict accordance with the recommendations in the Guide for the Care and Use of Laboratory Animals of the U.S. National Institutes of Health. Animal handling and experimental procedures were undertaken in compliance with the University of Pennsylvania's Institutional Animal Care and Use Committee (IACUC) guidelines (Animal Welfare Assurance Number: A3079-01).

### Reagents

PZQ, R(+)-verapamil HCl (dexverapamil), elacridar (GF120918, GG918), and MK 571 were from Sigma-Aldrich (St. Louis, MO). Tariquidar was from MedKoo Biosciences (Chapel Hill, NC). Ko143 was from Enzo Life Sciences (Farmingdale, NY), zosuquidar (LY335979) was from Toronto Research Chemicals (Toronto, ON), and Reversan was from Santa Cruz Biotechnology (Dallas, TX). Drugs were dissolved in dimethyl sulfoxide or ethanol for stock solutions, and were diluted to an appropriate concentration in culture media.

### Isolation of schistosomes

Female Swiss Webster mice infected with *S. mansoni* (NMRI strain) were provided by the Schistosomiasis Resource Center for distribution by BEI Resources, NIAID, NIH (*S. mansoni*, Strain NMRI - exposed Swiss Webster mice, NR-21963). Worms were perfused as described [71] at 6–7 weeks post-infection to obtain adult worms, or at 3–4 weeks post-infection to obtain juvenile worms. Perfused worms were maintained in schistosome medium [RPMI (Life Technologies, Grand Island, NY) plus 10% FBS (Sigma-Aldrich) and 1% penicillin/streptomycin (Sigma-Aldrich)] at 37°C and 5% CO_2_.

### Exposure of schistosomes to pharmacological compounds

For the drug treatments, 2–3 worm pairs per well were incubated overnight (up to 24 h) in a 12-well plate in schistosome medium with either 500 nM (adult) or 2 µM (juvenile) PZQ, in the presence or absence of inhibitors of ABC transporters. Following the drug treatments, worms were washed once with culture media and maintained and monitored for an additional 48 to 72 h in medium alone for the phenotypic analysis. In addition to testing ABC transporter inhibitors individually, we also assessed the effects of three combinations of inhibitors that would target multiple classes of the transporters. Combination A contained the Pgp (ABCB1) inhibitor [72] zosuqidar (10 µM), the BCRP (ABCG2) inhibitor [73] Ko143 (10 µM), and the MRP1 (ABCC1) inhibitor [30] MK 571 (25 µM). Combination B contained the Pgp/BCRP inhibitor [65], [66] elacridar (10 µM) and the MRP1 inhibitor [74] Reversan (20 µM). Combination C contained MK 571 (25 µM) and the Pgp inhibitor [75], [76] dexverapamil (20 µM). Dexverapamil is an enantiomer of verapamil with significantly less activity than the S(−) enantiomer against calcium channels, but which retains potent and selective competitive inhibitory activity against Pgp [77]. Control experiments contained carrier without drugs. There was some variation between different worm batches in their sensitivity to PZQ; in our experiments, we analyzed only those sets of schistosomes in which the large majority of worms remained motile following exposure to 500 nM PZQ.

### Analysis of BODIPY-PZQ retention by worms

Fluorescent (*R*)-PZQ-BODIPY was synthesized and purified as described previously [21], [78], and dissolved in DMSO. Though (*R*)-PZQ-BODIPY is active against schistosomes, it is far less potent (∼100-fold) than (R)-PZQ [78]. This reduced activity allowed us to use the probe without confounding effects from drug-induced contraction or paralysis. For testing of ABC transporter inhibitors, adult schistosomes were incubated overnight in schistosome medium plus 1 µM (*R*)-PZQ-BODIPY, in the presence or absence of the Pgp inhibitor dexverapamil (20 µM) or the MRP-1 inhibitor MK 571 (20 µM). Controls included worms incubated in schistosome medium alone (plus DMSO carrier), and worms incubated in transporter inhibitors alone. Indeed, this second control showed that incubation of schistosomes in tariquidar produced changes in worm fluorescence on its own, precluding the use of that compound in further experiments. Following the initial incubation, the medium was removed, and, following three washes, was replaced with schistosome medium without the (*R*)-PZQ-BODIPY, but still containing the transporter inhibitors that were included in the initial incubation. Fluorescence of live worms was observed at different time points following removal of the BODIPY-PZQ. Following 3 h of incubation, worms were fixed in 10% NBF (Sigma, St. Louis, MO), and photographed at identical exposures with a QImaging Qicam Fast 1394 digital camera on a Leica DMI3000B fluorescent microscope. For testing the effects of ABC transporter knockdown, siRNA-treated worms were incubated in schistosome medium for 3 days following electroporation with siRNAs. They were then incubated overnight in (*R*)-PZQ-BODIPY and processed in the same manner as the worms exposed to ABC transporter inhibitors.

For quantitation of fluorescence in the inhibitor-exposed worms, two regions of the worm were defined, and fluorescence intensity within corresponding regions of each worm was measured using Image J [79]. Data from both regions were essentially identical; the data presented in Fig. 4 are from one of these regions, slightly posterior to the oral sucker. We also examined fluorescence in worms incubated with 1 µM CellTracker Green BODIPY (Life Technologies, Inc.), a membrane-permeable form of BODIPY that is modified intracellularly to a membrane-impermeable form. For quantitation of fluorescence in worms subjected to RNAi (see below), the integrated intensity corresponding to the entire worm was measured using Image J and background intensity subtracted [80]. Plotted values are normalized to the mean control values. We also attempted to test the effects on retention of rhodamine-123, a fluorescent Pgp substrate, but control worms showed inconsistent results, and these experiments were not pursued further.

### RNA interference

Knockdown of RNAs encoding SMDR2 (NCBI Acc. #L26287), SmMRP1 (NCBI Acc. #GU967672), ABCA4 (Smp_056290), ABCB6 (Smp_134890), and MRP7/ABCC10 (Smp_147250) was as described [47], [81]. Briefly, following an overnight incubation in schistosome medium, adult worms (5 males plus 5 females) were placed in a 0.4 cm electroporation cuvette (USA Scientific, Ocala, FL) containing 50 µl siPORT (Life Technologies, Grand Island, NY) plus 3 µg of each of the siRNAs (IDT, Coralville, IA), either singly or in combination, targeting SMDR2, SmMRP1, MRP7, ABCA4, and ABCB6, or up to 15 µg luciferase siRNA (Life Technologies, Grand Island, NY; 3 µg per experimental siRNA used). The luciferase siRNA used for our control shows no significant similarity to any sequences from the *S. mansoni* gene database. siRNAs against the *S. mansoni* transporters were designed using the IDT SciTools RNAi Design server; sequences and targets are listed in Table 1. Worms were electroporated in this solution with a 20 ms square-wave pulse of 125 volts (BioRad Pulser XCell). Following electroporation, worms were incubated in schistosome medium for 2 days. They were then sorted into 2–3 males/female pairs per well in a 12-well plate, in which they were subsequently incubated in schistosome medium with carrier alone, or in medium plus PZQ (800 nM), as described above, and subsequently analyzed for motility.

**Table 1:** Target genes and siRNA sequences used for the RNA interference experiments.

Target gene	siRNA sequence (5′-3′)	Target region (bp)
**SMDR2**	TCGATCAAACCAACCAATCTCCTGTTT	2332–2358
**SmMRP1**	GACCAATCAGCTAACCATAAATTTGTT	3834–3860
**ABCA4**	CTATTAACTGGTAGAGAAACACTTA	5137–5161
**ABCB6**	TCAGCTGATCGAACAGATATTGAAC	421–445
**MRP7**	GACCAATCAGCTAACCATAAATTTGTT	3834–3860

### Worm motility analysis

Schistosomes were observed using a Leica stereomicroscope and digitally recorded (QImaging Qicam Fast 1394). These video recordings were then used to analyze motility of individual worms over 5- or 10-second time spans using MaxTraq-Lite+ motion analysis software (Innovision Systems, Columbiaville, MO). We used the change in distance between the head and tail of individual worms as an estimate of motility (as the worm moves, this distance will change). Head-tail distance was measured every 0.3 s by calculating the distance between digitized markers placed manually at the same points on the worm in each frame analyzed. The amount by which the measured distance changed from its previous value was calculated for each measurement, and the average change in head-tail distance was then averaged for each worm and normalized to control values. To correct for digitizing errors, the same process was used for dead worms, and the measured average motility (which theoretically should be 0) was subtracted as “noise” from the experimental values. Other measures of motility (changes in angle between anterior and posterior ends of the worm or edge-based tracking by adapting algorithms in the program Cell Track [82]) produced similar results (not shown). Based on controls using 20 µM PZQ (to completely paralyze the worms; Fig. S4) or dead worms, it is clear that this method is valid for measuring inhibition of activity. On the other hand, it does not appear to be as effective in registering hyperactivity (see Fig. S4), in which evaluating changes in head-tail distance may not account for increases in spasticity, twitching, or peristalsis, none of which would be likely to change head-tail distance. In the experiments here, we measured only decreases in motility. We found no significant differences in motility between male and female worms under any of the test conditions; therefore all data presented combine results for both sexes. For all experiments, several independent wells (≥3) were tested for each condition. All experiments were also repeated with worms perfused from at least two independent batches of infected mice at different times. Motility of individual worms was assessed (numbers of worms tested are provided in the figure legends), and analyzed as described.

### Real-time RT-PCR

Total RNA was extracted as described [44], using RNAqueous-4-PCR (Life Technologies) and subsequently treated with Turbo-DNAase (Life Technologies) according to the manufacturer's instructions. Two step qRT-PCR was employed to measure RNAi knockdown. First-Strand cDNA was synthesized using the SMARTer cDNA synthesis kit (Clontech, Mt. View, CA) and amplified using the Brilliant II SYBR green qRT-PCR Master kit (Agilent Technologies, Santa Clara, CA) on an Applied Biosystems 3500 Genetic Analyzer according to the manufacturer's recommendations (omitting the cDNA synthesis step). Primers used for the amplification of SMDR2, SmMRP1 and 18S ribosomal RNA have been described previously [43], [44]. Primers used for amplification of ABCA4 (Smp_056290) were: ABCA4-Sy1 (5′- GGGTGGTATGACAACAGCAA -3′) and ABCA4-Sy2 (5′- GAGCTGAAATTGGCCCTCTA -3′). Primers used for amplification of ABCB6 (Smp_134890) were: ABCB6-Sy1 (5′- TGCTATTGCCGCTGACATAC -3′) and ABCB6-Sy2 (5′- CCAATGCTGATGTAGCTTCG -3′). Primers used for amplification of MRP7 (Smp_147250) were: MRP7-Sy1 (5′- AGCTGGTGGGAGCAGTCTTA -3′) and MRP7-Sy2 (5′- ATCCAACTGGTGTGTGACGA -3′). Data were analyzed using the 2^−ΔΔ^C_t_ method [83] to determine the relative expression ratio between target (transporters) and reference genes (18S RNA).

### Analysis of tegumental disruption

Adult male schistosomes were fixed overnight with 10% NBF, and morphological changes were examined on a Leica bright field microscope using a 20× objective. Images were acquired on a QICAM (QImaging, Surrey, BC) digital camera in an equivalent area near the oral sucker region, to establish consistency of observation for all the samples. Images were analyzed with QCapture Pro 7 software (QImaging) or commercial image processing software (eg, Canvas, ACD Systems).

### Statistics

Data were analyzed with GraphPad Prism or Excel, expressed as arithmetic means ± SEM, and tested for statistical significance using statistical tests noted in the figure legends.

## Supporting Information

Figure S1 **ABC transporter inhibitors other than tariquidar show no obvious effects on adult schistosome tegumental integrity in the absence of PZQ.** Adult schistosomes were treated as described, and an equivalent tegumental region just below the oral sucker of male worms analyzed by optical microscopy, as in Fig. 2. (**A**) 0.5% DMSO (Control). (**B**) Combination A. (**C**) Combination B. (**D**) 10 µM tariquidar.
(TIF)

Figure S2 **Neither dexverapamil nor MK 571 increase measured fluorescence intensity in adult male worms exposed to BODIPY that is not conjugated to PZQ.** Schistosomes were exposed as in Fig. 4 to 1 µM CellTracker Green BODIPY in the presence or absence of 20 µM dexverapamil or 20 µM MK 571, and fluorescence intensity measured, also as described in Fig. 4. Black bars represent worms incubated in CellTracker Green BODIPY, with added dexverapamil (Dex; n = 4), MK 571 (n = 3), or DMSO carrier (Control; n = 4); white bars represent worms incubated without added BODIPY, but with dexverapamil (Dex; n = 3), MK 571 (n = 3), or DMSO carrier (Control; n = 3). ANOVA with Dunnett's Multiple Comparison post test shows no significant difference between the BODIPY Control and the BODIPY plus dexverapamil or MK 571.
(TIF)

Figure S3 **Simultaneous knockdown of 5 multidrug transporters using siRNAs in** ***S. mansoni*** **adults.** Expression levels of RNAs encoding the targeted ABC transporters were determined by qRT-PCR using gene-specific primers and SYBR green (see Materials and Methods). Adult schistosomes were electroporated with a combination of siRNAs targeting 5 different ABC transporters (3 µg each, 15 µg total; clear bars); control worms were electroporated with 15 µg siRNA targeting luciferase (black bars). The text below the X-axis indicates the transcript that was quantified. The targeted transporters were: SMDR2 (L26287; n = 5); SmMRP1 (Smp_171740; n = 5); ABCA4 (Smp_056290; n = 3), ABCB6 (Smp_134890; n = 3), and SmMRP7 (ABCC10, Smp_147250; n = 3). ** and *** indicate P<0.01 and P<0.001, respectively, t-test.
(TIF)

Figure S4 **Validation of motility assay for worm paralysis.** Worms were incubated in either 20 µM PZQ or 40 µM serotonin (which causes hyperactivity), and motility measured as described in Materials and Methods. PZQ paralyzed the worms, which is confirmed by the motility assay. In contrast, though serotonin clearly caused hyperactivity when worms were examined visually, the motility assay did not show a significant increase in activity, likely reflecting the inability of this assay to measure increases in movements such as twitching and peristalsis that do not necessarily change head-tail distance. n = 3, ** indicates P<0.01, compared with control worms, ANOVA with Dunnett's Multiple Comparison post test.
(TIF)

## References

[1] Hotez PJ, Fenwick A (2009) Schistosomiasis in Africa: an emerging tragedy in our new global health decade. PLoS Neglected Tropical Diseases 3: e485. 19787054 10.1371/journal.pntd.0000485 PMC2746322

[2] King CH, Dangerfield-Cha M (2008) The unacknowledged impact of chronic schistosomiasis. Chronic Illness 4: 65–79. 18322031 10.1177/1742395307084407

[3] van der Werf MJ, de Vlas SJ, Brooker S, Looman CW, Nagelkerke NJ, et al (2003) Quantification of clinical morbidity associated with schistosome infection in sub-Saharan Africa. Acta Tropica 86: 125–139. 12745133 10.1016/s0001-706x(03)00029-9

[4] Doenhoff MJ, Pica-Mattoccia L (2006) Praziquantel for the treatment of schistosomiasis: its use for control in areas with endemic disease and prospects for drug resistance. Expert Review of Anti-infective Therapy 4: 199–210. 16597202 10.1586/14787210.4.2.199

[5] Hagan P, Appleton CC, Coles GC, Kusel JR, Tchuem-Tchuente LA (2004) Schistosomiasis control: keep taking the tablets. Trends in Parasitology 20: 92–97. 14747023 10.1016/j.pt.2003.11.010

[6] Caffrey CR (2007) Chemotherapy of schistosomiasis: present and future. Current Opinion in Chemical Biology 11: 433–439. 17652008 10.1016/j.cbpa.2007.05.031

[7] Toure S, Zhang Y, Bosque-Oliva E, Ky C, Ouedraogo A, et al (2008) Two-year impact of single praziquantel treatment on infection in the national control programme on schistosomiasis in Burkina Faso. Bulletin of the World Health Organization 86: 780–787. 18949215 10.2471/BLT.07.048694 PMC2649514

[8] Vennervald BJ, Booth M, Butterworth AE, Kariuki HC, Kadzo H, et al (2005) Regression of hepatosplenomegaly in Kenyan school-aged children after praziquantel treatment and three years of greatly reduced exposure to *Schistosoma mansoni* . Transactions of the Royal Society of Tropical Medicine and Hygiene 99: 150–160. 15607341 10.1016/j.trstmh.2004.06.009

[9] Fenwick A, Savioli L, Engels D, Robert Bergquist N, Todd MH (2003) Drugs for the control of parasitic diseases: current status and development in schistosomiasis. Trends in Parasitology 19: 509–515. 14580962 10.1016/j.pt.2003.09.005

[10] Redman CA, Robertson A, Fallon PG, Modha J, Kusel JR, et al (1996) Praziquantel: an urgent and exciting challenge. Parasitology Today 12: 14–20. 15275303 10.1016/0169-4758(96)80640-5

[11] Greenberg RM (2005) Are Ca*^2+^* channels targets of praziquantel action? International Journal for Parasitology 35: 1–9. 15619510 10.1016/j.ijpara.2004.09.004

[12] Doenhoff MJ, Cioli D, Utzinger J (2008) Praziquantel: mechanisms of action, resistance and new derivatives for schistosomiasis. Current Opinion in Infectious Diseases 21: 659–667. 18978535 10.1097/QCO.0b013e328318978f

[13] Mutapi F, Rujeni N, Bourke C, Mitchell K, Appleby L, et al (2011) *Schistosoma haematobium* treatment in 1–5 year old children: safety and efficacy of the antihelminthic drug praziquantel. PLoS Neglected Tropical Diseases 5: e1143. 21610855 10.1371/journal.pntd.0001143 PMC3096601

[14] Behbehani M, Savioli L (1998) Report of the WHO informal consultation on monitoring of drug efficacy in the control of schistosomiasis and intestinal nematodes. Geneva: World Health Organization. WHO/CDS/CPC/SIP/99.1.

[15] Tukahebwa E, Vennervald BJ, Nuwaha F, Kabatereine NB, Magnussen P (2013) Comparative efficacy of one versus two doses of praziquantel on cure rate of *Schistosoma mansoni* infection and re-infection in Mayuge District, Uganda. Transactions of the Royal Society of Tropical Medicine and Hygiene 107: 397–404. 23596262 10.1093/trstmh/trt024

[16] Melman SD, Steinauer ML, Cunningham C, Kubatko LS, Mwangi IN, et al (2009) Reduced susceptibility to praziquantel among naturally occurring Kenyan isolates of *Schistosoma mansoni* . PLoS Neglected Tropical Diseases 3: e504. 19688043 10.1371/journal.pntd.0000504 PMC2721635

[17] Couto FF, Coelho PM, Araujo N, Kusel JR, Katz N, et al (2011) *Schistosoma mansoni*: a method for inducing resistance to praziquantel using infected *Biomphalaria glabrata* snails. Memorias do Instituto Oswaldo Cruz 106: 153–157. 21537673 10.1590/s0074-02762011000200006

[18] Fallon PG, Doenhoff MJ (1994) Drug-resistant schistosomiasis: resistance to praziquantel and oxamniquine induced in *Schistosoma mansoni* in mice is drug specific. American Journal of Tropical Medicine and Hygiene 51: 83–88. 8059919 10.4269/ajtmh.1994.51.83

[19] Greenberg RM (2013) New approaches for understanding mechanisms of drug resistance in schistosomes. Parasitology 140: 1534–1546. 23552512 10.1017/S0031182013000231 PMC3775338

[20] Day TA, Botros S (2006) Drug resistance in schistosomes. In: Maule A, Marks NJ, editors. Parasitic Flatworms: Molecular Biology, Biochemistry, Immunology and Physiology. Oxfordshire, UK: CAB International. pp. 256–268.

[21] Aragon AD, Imani RA, Blackburn VR, Cupit PM, Melman SD, et al (2009) Towards an understanding of the mechanism of action of praziquantel. Molecular and Biochemical Parasitology 164: 57–65. 19100294 10.1016/j.molbiopara.2008.11.007 PMC2886009

[22] Xiao SH, Catto BA, Webster LT Jr (1985) Effects of praziquantel on different developmental stages of *Schistosoma mansoni in vitro* and *in vivo* . Journal of Infectious Diseases 151: 1130–1137. 3998507 10.1093/infdis/151.6.1130

[23] Sabah AA, Fletcher C, Webbe G, Doenhoff MJ (1986) *Schistosoma mansoni*: chemotherapy of infections of different ages. Experimental Parasitology 61: 294–303. 3086114 10.1016/0014-4894(86)90184-0

[24] Pica-Mattoccia L, Cioli D (2004) Sex- and stage-related sensitivity of *Schistosoma mansoni* to *in vivo* and *in vitro* praziquantel treatment. International Journal for Parasitology 34: 527–533. 15013742 10.1016/j.ijpara.2003.12.003

[25] Lespine A, Alvinerie M, Vercruysse J, Prichard RK, Geldhof P (2008) ABC transporter modulation: a strategy to enhance the activity of macrocyclic lactone anthelmintics. Trends in Parasitology 24: 293–298. 18514030 10.1016/j.pt.2008.03.011

[26] Liang XJ, Aszalos A (2006) Multidrug transporters as drug targets. Current Drug Targets 7: 911–921. 16918320 10.2174/138945006778019264

[27] Pohl A, Lage H, Muller P, Pomorski T, Herrmann A (2002) Transport of phosphatidylserine via MDR1 (multidrug resistance 1) P-glycoprotein in a human gastric carcinoma cell line. Biochemical Journal 365: 259–268. 12071854 10.1042/BJ20011880 PMC1222671

[28] Aye ILMH, Singh AT, Keelan JA (2009) Transport of lipids by ABC proteins: interactions and implications for cellular toxicity, viability, and function. Chemico-Biological Interactions 180: 327–339. 19426719 10.1016/j.cbi.2009.04.012

[29] Sundaram P, Echalier B, Han W, Hull D, Timmons L (2006) ATP-binding cassette transporters are required for efficient RNA interference in *Caenorhabditis elegans* . Molecular Biology of the Cell 17: 3678–3688. 16723499 10.1091/mbc.E06-03-0192 PMC1525249

[30] Leier I, Jedlitschky G, Buccholz U, Cole SP, Deeley RG, et al (1994) The MRP gene encodes an ATP-dependent export pump for leukotriene C4 and structurally related conjugates. Journal of Biological Chemistry 269: 27807–27810. 7961706

[31] Johnstone RW, Ruefli AA, Smyth MJ (2000) Multiple physiological functions for multidrug transporter P-glycoprotein? Trends in Biochemical Sciences 25: 1–6. 10637601 10.1016/s0968-0004(99)01493-0

[32] Mizutani T, Masuda M, Nakai E, Furumiya K, Togawa H, et al (2008) Genuine functions of P-glycoprotein (ABCB1). Current Drug Metabolism 9: 167–174. 18288958 10.2174/138920008783571756

[33] Yabe T, Suzuki N, Furukawa T, Ishihara T, Katsura I (2005) Multidrug resistance-associated protein MRP-1 regulates dauer diapause by its export activity in *Caenorhabditis elegans* . Development 132: 3197–3207. 15983401 10.1242/dev.01909

[34] Johnstone RW, Ruefli AA, Tainton KM, Smyth MJ (2000) A role for P-glycoprotein in regulating cell death. Leukemia and Lymphoma 38: 1–11. 10811443 10.3109/10428190009060314

[35] Baguley BC (2010) Multidrug resistance in cancer. Methods in Molecular Biology 596: 1–14. 19949917 10.1007/978-1-60761-416-6_1

[36] van de Ven R, Oerlemans R, van der Heijden JW, Scheffer GL, de Gruijl TD, et al (2009) ABC drug transporters and immunity: novel therapeutic targets in autoimmunity and cancer. Journal of Leukocyte Biology 86: 1075–1087. 19745159 10.1189/jlb.0309147

[37] Gottesman MM, Ling V (2006) The molecular basis of multidrug resistance in cancer: the early years of P-glycoprotein research. FEBS Letters 580: 998–1009. 16405967 10.1016/j.febslet.2005.12.060

[38] James CE, Hudson AL, Davey MW (2009) Drug resistance mechanisms in helminths: is it survival of the fittest? Trends in Parasitology 25: 328–335. 19541539 10.1016/j.pt.2009.04.004

[39] Lespine A, Menez C, Bourguinat C, Prichard RK (2012) P-glycoproteins and other multidrug resistance transporters in the pharmacology of anthelmintics: prospects for reversing transport-dependent anthelmintic resistance. International Journal for Parasitology: Drugs and Drug Resistance 2: 58–75. 24533264 10.1016/j.ijpddr.2011.10.001 PMC3862436

[40] Ardelli BF (2013) Transport proteins of the ABC systems superfamily and their role in drug action and resistance in nematodes. Parasitology International 62: 639–646. 23474412 10.1016/j.parint.2013.02.008

[41] Greenberg RM (2013) ABC multidrug transporters in schistosomes and other parasitic flatworms. Parasitology International 62: 647–653. 23474413 10.1016/j.parint.2013.02.006 PMC3726539

[42] Kasinathan RS, Greenberg RM (2012) Pharmacology and potential physiological significance of schistosome multidrug resistance transporters. Experimental Parasitology 132: 2–6. 21420955 10.1016/j.exppara.2011.03.004 PMC3154572

[43] Messerli SM, Kasinathan RS, Morgan W, Spranger S, Greenberg RM (2009) *Schistosoma mansoni* P-glycoprotein levels increase in response to praziquantel exposure and correlate with reduced praziquantel susceptibility. Molecular and Biochemical Parasitology 167: 54–59. 19406169 10.1016/j.molbiopara.2009.04.007 PMC2694853

[44] Kasinathan RS, Morgan WM, Greenberg RM (2010) *Schistosoma mansoni* express higher levels of multidrug resistance-associated protein 1 (SmMRP1) in juvenile worms and in response to praziquantel. Molecular and Biochemical Parasitology 173: 25–31. 20470831 10.1016/j.molbiopara.2010.05.003 PMC2896741

[45] Hines-Kay J, Cupit PM, Sanchez MC, Rosenberg GH, Hanelt B, et al (2012) Transcriptional analysis of *Schistosoma mansoni* treated with praziquantel *in vitro* . Molecular and Biochemical Parasitology 186: 87–94. 23022771 10.1016/j.molbiopara.2012.09.006 PMC3513576

[46] Kasinathan RS, Goronga T, Messerli SM, Webb TR, Greenberg RM (2010) Modulation of a *Schistosoma mansoni* multidrug transporter by the antischistosomal drug praziquantel. FASEB Journal 24: 128–135. 19726755 10.1096/fj.09-137091 PMC2797036

[47] Kasinathan RS, Morgan WM, Greenberg RM (2011) Genetic knockdown and pharmacological inhibition of parasite multidrug resistance transporters disrupts egg production in *Schistosoma mansoni* . PLoS Neglected Tropical Diseases 5: e1425. 22163059 10.1371/journal.pntd.0001425 PMC3232217

[48] Sato H, Kusel JR, Thornhill J (2004) Excretion of fluorescent substrates of mammalian multidrug resistance-associated protein (MRP) in the *Schistosoma mansoni* excretory system. Parasitology 128: 43–52. 15002903 10.1017/s0031182003004177

[49] Sato H, Kusel JR, Thornhill J (2002) Functional visualization of the excretory system of adult *Schistosoma mansoni* by the fluorescent marker resorufin. Parasitology 125: 527–535. 12553571 10.1017/s0031182002002536

[50] Roe M, Folkes A, Ashworth P, Brumwell J, Chima L, et al (1999) Reversal of P-glycoprotein mediated multidrug resistance by novel anthranilamide derivatives. Bioorganic and Medicinal Chemistry Letters 9: 595–600. 10098671 10.1016/s0960-894x(99)00030-x

[51] Mistry P, Stewart AJ, Dangerfield W, Okiji S, Liddle C, et al (2001) *In vitro* and *in vivo* reversal of P-glycoprotein-mediated multidrug resistance by a novel potent modulator, XR9576. Cancer Research 61: 749–758. 11212278

[52] Fox E, Bates SE (2007) Tariquidar (XR9576): a P-glycoprotein drug efflux pump inhibitor. Expert Review of Anti-Cancer Therapy 7: 447–459. 10.1586/14737140.7.4.447 17428165

[53] Martin C, Berridge G, Mistry P, Higgins C, Charlton P, et al (1999) The molecular interaction of the high affinity reversal agent XR9576 with P-glycoprotein. British Journal of Pharmacology 128: 403–411. 10510451 10.1038/sj.bjp.0702807 PMC1571648

[54] Mehlhorn H, Becker B, Andrews P, Thomas H, Frenkel JK (1981) *In vivo* and *in vitro* experiments on the effects of praziquantel on *Schistosoma mansoni*: a light and electron microscopic study. Arzneimittel-Forschung 31: 544–554. 7195245

[55] Xiao SH, Catto BA (1989) Comparative *in vitro* and *in vivo* activity of racemic praziquantel and its levorotated isomer on *Schistosoma mansoni* . Journal of Infectious Diseases 159: 589–592. 2915173 10.1093/infdis/159.3.589

[56] Andrews P, Thomas H, Pohlke R, Seubert J (1983) Praziquantel. Medicinal Research Reviews 3: 147–200. 6408323 10.1002/med.2610030204

[57] Tanaka M, Ohmae H, Utsunomiya H, Nara T, Irie Y, et al (1989) A comparison of the antischistosomal effect of levo- and dextro-praziquantel on *Schistosoma japonicum* and *S. mansoni* in mice. American Journal of Tropical Medicine and Hygiene 41: 198–203. 2505624 10.4269/ajtmh.1989.41.198

[58] Woelfle M, Seerden JP, de Gooijer J, Pouwer K, Olliaro P, et al (2011) Resolution of praziquantel. PLoS Neglected Tropical Diseases 5: e1260. 21949890 10.1371/journal.pntd.0001260 PMC3176743

[59] Kongs A, Marks G, Verle P, Van der Stuyft P (2008) The unreliability of the Kato-Katz technique limits its usefulness for evaluating *S. mansoni* infections. Tropical Medicine and International Health 6: 163–169. 10.1046/j.1365-3156.2001.00687.x 11299032

[60] Lin DD, Liu JX, Liu YM, Hu F, Zhang YY, et al (2008) Routine Kato–Katz technique underestimates the prevalence of *Schistosoma japonicum*: a case study in an endemic area of the People's Republic of China. Parasitology International 57: 281–286. 18485807 10.1016/j.parint.2008.04.005

[61] Lodh N, Mwansa JCL, Mutengo MM, Shiff CJ (2013) Diagnosis of *Schistosoma mansoni* without the stool: detecting DNA from filtered urine comparison of three diagnostic tests to detect *Schistosoma mansoni* infection from filtered urine in Zambia. American Journal of Tropical Medicine and Hygiene 89: 46–50. 23716406 10.4269/ajtmh.13-0104 PMC3748486

[62] Higgins CF, Gottesman MM (1992) Is the multidrug transporter a flippase? Trends in Biochemical Sciences 17: 18–21. 1374941 10.1016/0968-0004(92)90419-a

[63] Sharom FJ (2011) The P-glycoprotein multidrug transporter. Essays in Biochemistry 50: 161–178. 21967057 10.1042/bse0500161

[64] Kannan P, Telu S, Shukla S, Ambudkar SV, Pike VW, et al (2011) The “specific” P-glycoprotein inhibitor tariquidar is also a substrate and an inhibitor for Breast Cancer Resistance Protein (BCRP/ABCG2). ACS Chemical Neuroscience 2: 82–89. 22778859 10.1021/cn100078a PMC3369725

[65] Hyafil F, Vergely C, Du Vignaud P, Grand-Perret T (1993) *In vitro* and *in vivo* reversal of multidrug resistance by GF120918, an acridonecarboxamide derivative. Cancer Research 53: 4595–4602. 8402633

[66] de Bruin M, Miyake K, Litman T, Robey R, Bates SE (1999) Reversal of resistance by GF120918 in cell lines expressing the ABC half-transporter, MXR. Cancer Letters 146: 117–126. 10656616 10.1016/s0304-3835(99)00182-2

[67] Savage J, Meaney M, Brennan GP, Hoey E, Trudgett A, et al (2013) Effect of the P-glycoprotein inhibitor, R(+)-verapamil on the drug susceptibility of a triclabendazole-resistant isolate of *Fasciola hepatica* . Veterinary Parasitology 195: 72–86. 23597772 10.1016/j.vetpar.2013.03.007

[68] Walter M, Kuris A (2003) Methods for the inhibition of egg production in trematodes. US Patent Number 6,514,963 B2.

[69] Hayeshi R, Masimirembwa C, Mukanganyama S, Ungell AL (2006) The potential inhibitory effect of antiparasitic drugs and natural products on P-glycoprotein mediated efflux. European Journal of Pharmaceutical Sciences 29: 70–81. 16846720 10.1016/j.ejps.2006.05.009

[70] Gray DJ, McManus DP, Li Y, Williams GM, Bergquist R, et al (2010) Schistosomiasis elimination: lessons from the past guide the future. Lancet Infectious Diseases 10: 733–736. 20705513 10.1016/S1473-3099(10)70099-2

[71] Lewis FA (1998) Schistosomiasis. In: Coligan JE, Kruisbeek AM, Margulies DH, Shevach EM, Strober W et al.., editors. Current Protocols in Immunology. pp. 19.11.11–19.11.28.

[72] Dantzig AH, Shepard RL, Cao J, Law KL, Ehlhardt WJ, et al (1996) Reversal of P-glycoprotein-mediated multidrug resistance by a potent cyclopropyldibenzosuberane modulator, LY335979. Cancer Research 56: 4171–4179. 8797588

[73] Allen JD, van Loevezijn A, Lakhai JM, van der Valk M, van Tellingen O, et al (2002) Potent and specific inhibition of the breast cancer resistance protein multidrug transporter *in vitro* and in mouse intestine by a novel analogue of fumitremorgin C. Molecular Cancer Therapeutics 1: 417–425. 12477054

[74] Burkhart CA, Watt F, Murray J, Pajic M, Prokvolit A, et al (2009) Small molecule MRP1 inhibitor Reversan increases the therapeutic index of chemotherapy in mouse model of neuroblastoma. Cancer Research 69: 6573–6580. 19654298 10.1158/0008-5472.CAN-09-1075 PMC2746061

[75] Gruber A, Peterson C, Reizenstein P (1988) D-verapamil and L-verapamil are equally effective in increasing vincristine accumulation in leukemic cells *in vitro* . International Journal of Cancer 41: 224–226. 3422225 10.1002/ijc.2910410211

[76] Pirker R, Keilhauer G, Raschack M, Lechner C, Ludwig H (1990) Reversal of multi-drug resistance in human KB cell lines by structural analogs of verapamil. International Journal of Cancer 45: 916–919. 2335394 10.1002/ijc.2910450523

[77] Varma MV, Ashokraj Y, Dey CS, Panchagnula R (2003) P-glycoprotein inhibitors and their screening: a perspective from bioavailability enhancement. Pharmacological Research 48: 347–359. 12902205 10.1016/s1043-6618(03)00158-0

[78] Sharma LK, Cupit PM, Goronga T, Webb TR, Cunningham C (2014) Design and synthesis of molecular probes for the determination of the target of the anthelmintic drug praziquantel. Bioorganic and Medicinal Chemistry Letters 24: 2469–2472. 24775301 10.1016/j.bmcl.2014.04.014 PMC4055354

[79] Schneider CA, Rasband WS, Elicieri KW (2012) NIH Image to ImageJ: 25 years of image analysis. Nature Methods 9: 671–675. 22930834 10.1038/nmeth.2089 PMC5554542

[80] Gavet O, Pines J (2010) Progressive activation of CyclinB1-Cdk1 coordinates entry to mitosis. Developmental Cell 18: 533–543. 20412769 10.1016/j.devcel.2010.02.013 PMC3325599

[81] Krautz-Peterson G, Radwanska M, Ndegwa D, Shoemaker CB, Skelly PJ (2007) Optimizing gene suppression in schistosomes using RNA interference. Molecular and Biochemical Parasitology 153: 194–202. 17420062 10.1016/j.molbiopara.2007.03.006

[82] Sacan A, Ferhatosmanoglu H, Coskun H (2008) CellTrack: an open-source software for cell tracking and motility analysis. Bioinformatics 24: 1647–1649. 18511469 10.1093/bioinformatics/btn247

[83] Schmittgen TD, Livak KJ (2008) Analyzing real-time PCR data by the comparative C_T_ method. Nature Protocols 3: 1101–1108. 18546601 10.1038/nprot.2008.73

