# Supplementary figures and images for: Inhibition or Knockdown of ABC Transporters Enhances Susceptibility of Adult and Juvenile Schistosomes to Praziquantel
Source: PLoS Negl Trop Dis. 2014 Oct 16;8(10):e3265. doi: 10.1371/journal.pntd.0003265 (PMC4199547; doi:10.1371/journal.pntd.0003265)

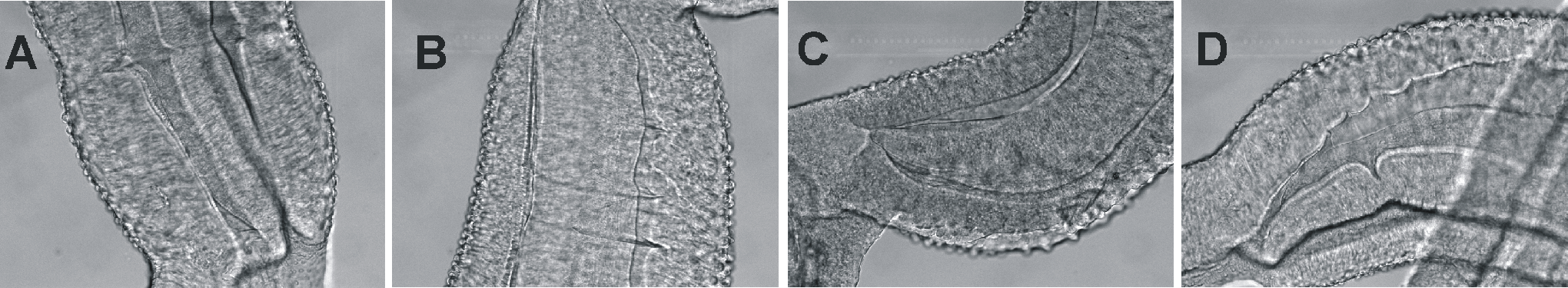

Supplement: Figure S1 — ABC transporter inhibitors other than tariquidar show no obvious effects on adult schistosome tegumental integrity in the absence of PZQ. Adult schistosomes were treated as described, and an equivalent tegumental region just below the oral sucker of male worms analyzed by optical microscopy, as in Fig. 2. (A) 0.5% DMSO (Control). (B) Combination A. (C) Combination B. (D) 10 µM tariquidar. (TIF) [file pntd.0003265.s001.tif]

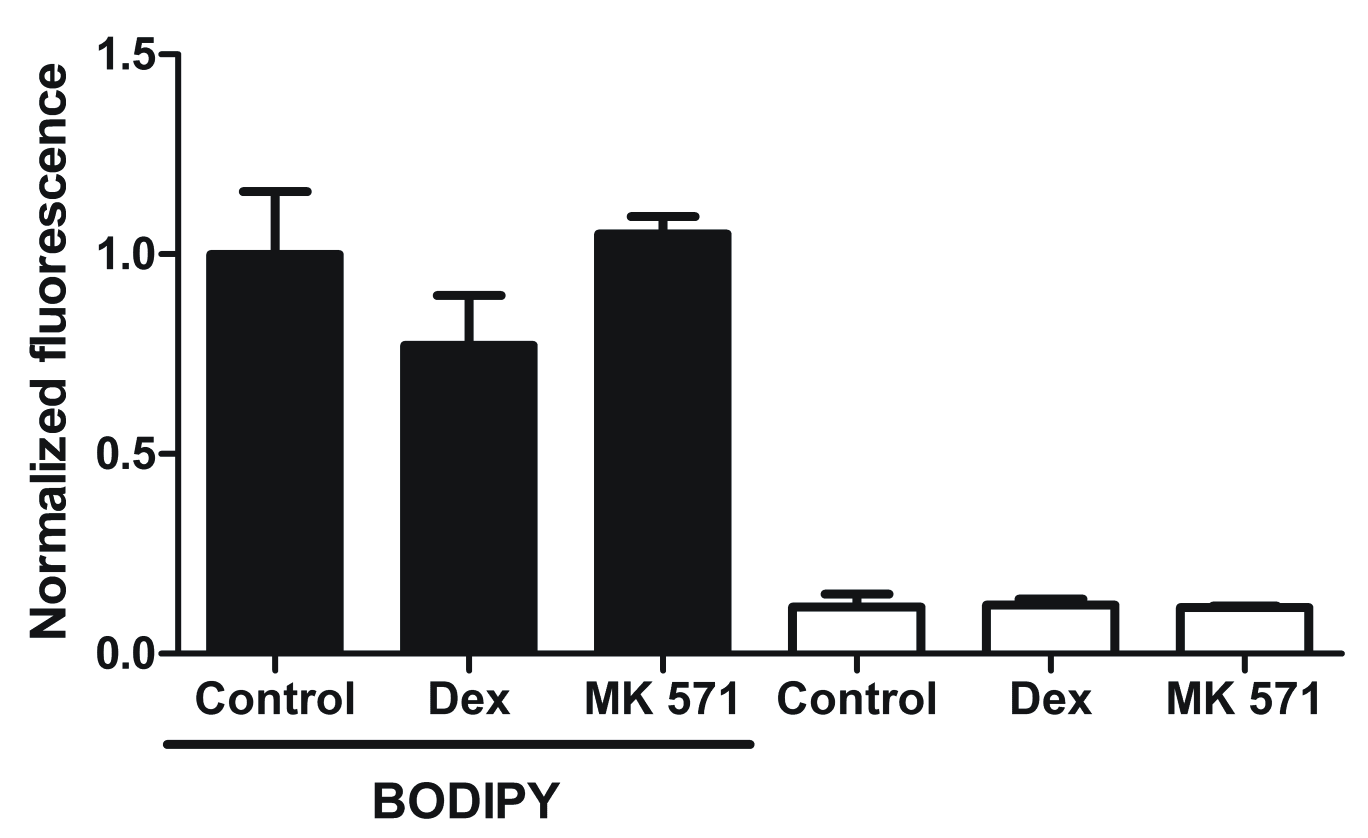

Supplement: Figure S2 — Neither dexverapamil nor MK 571 increase measured fluorescence intensity in adult male worms exposed to BODIPY that is not conjugated to PZQ. Schistosomes were exposed as in Fig. 4 to 1 µM CellTracker Green BODIPY in the presence or absence of 20 µM dexverapamil or 20 µM MK 571, and fluorescence intensity measured, also as described in Fig. 4. Black bars represent worms incubated in CellTracker Green BODIPY, with added dexverapamil (Dex; n = 4), MK 571 (n = 3), or DMSO carrier (Control; n = 4); white bars represent worms incubated without added BODIPY, but with dexverapamil (Dex; n = 3), MK 571 (n = 3), or DMSO carrier (Control; n = 3). ANOVA with Dunnett's Multiple Comparison post test shows no significant difference between the BODIPY Control and the BODIPY plus dexverapamil or MK 571. (TIF) [file pntd.0003265.s002.tif]

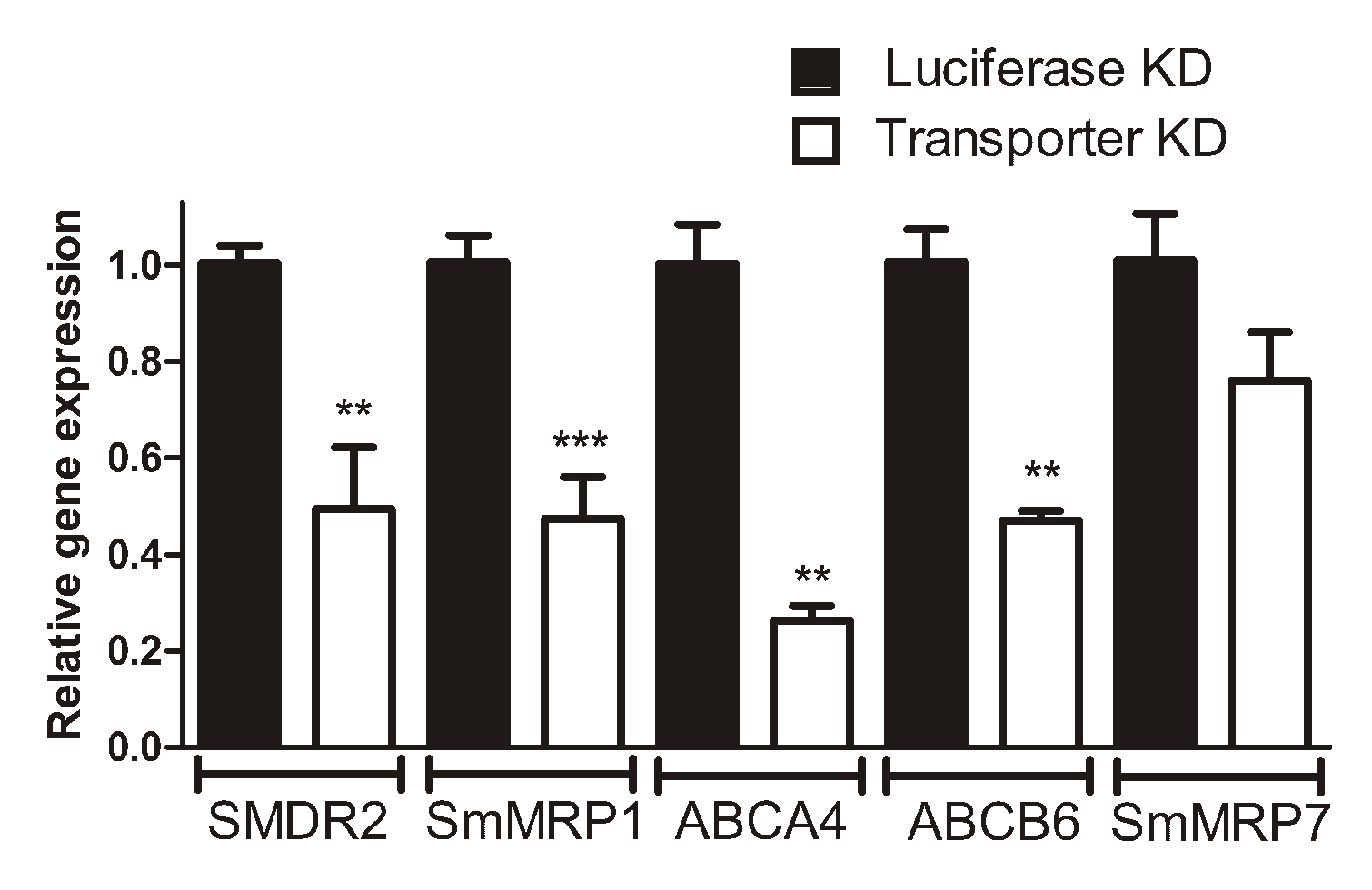

Supplement: Figure S3 — Simultaneous knockdown of 5 multidrug transporters using siRNAs in S. mansoni adults. Expression levels of RNAs encoding the targeted ABC transporters were determined by qRT-PCR using gene-specific primers and SYBR green (see Materials and Methods). Adult schistosomes were electroporated with a combination of siRNAs targeting 5 different ABC transporters (3 µg each, 15 µg total; clear bars); control worms were electroporated with 15 µg siRNA targeting luciferase (black bars). The text below the X-axis indicates the transcript that was quantified. The targeted transporters were: SMDR2 (L26287; n = 5); SmMRP1 (Smp_171740; n = 5); ABCA4 (Smp_056290; n = 3), ABCB6 (Smp_134890; n = 3), and SmMRP7 (ABCC10, Smp_147250; n = 3). ** and *** indicate P<0.01 and P<0.001, respectively, t-test. (TIF) [file pntd.0003265.s003.tif]

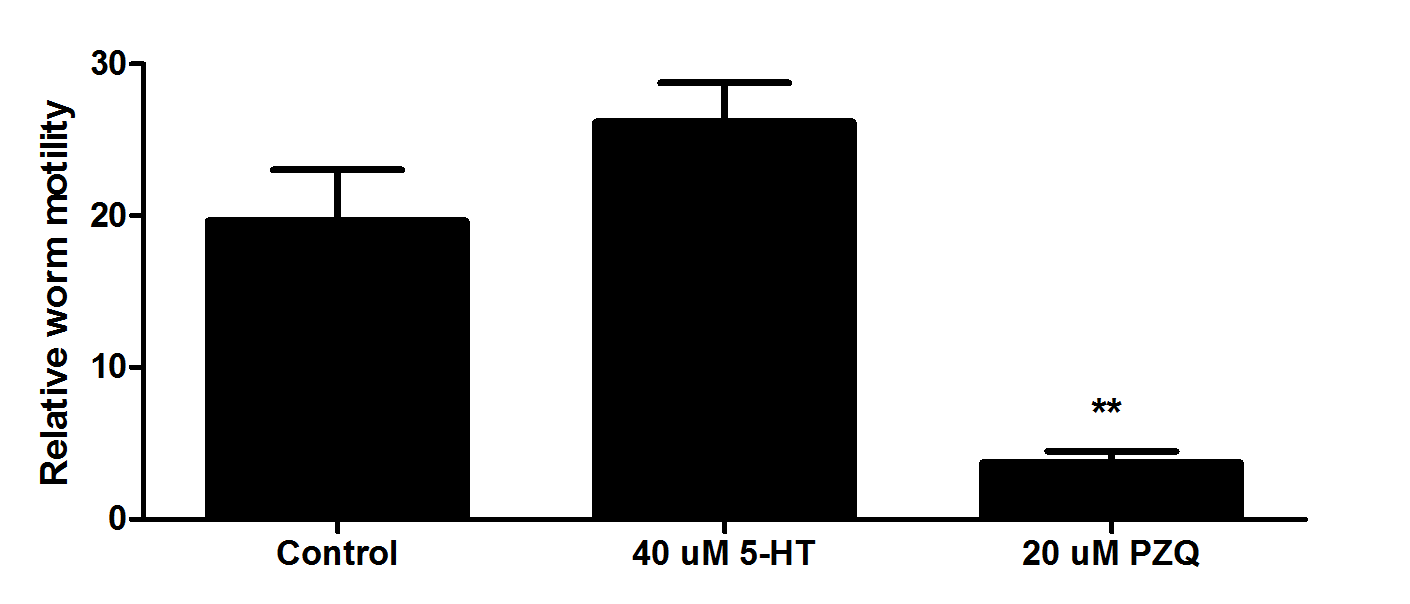

Supplement: Figure S4 — Validation of motility assay for worm paralysis. Worms were incubated in either 20 µM PZQ or 40 µM serotonin (which causes hyperactivity), and motility measured as described in Materials and Methods. PZQ paralyzed the worms, which is confirmed by the motility assay. In contrast, though serotonin clearly caused hyperactivity when worms were examined visually, the motility assay did not show a significant increase in activity, likely reflecting the inability of this assay to measure increases in movements such as twitching and peristalsis that do not necessarily change head-tail distance. n = 3, ** indicates P<0.01, compared with control worms, ANOVA with Dunnett's Multiple Comparison post test. (TIF) [file pntd.0003265.s004.tif]
